# From the Synthesis of Nanovehicles to Participation in the First Nanocar Race—View from the French Team

**DOI:** 10.3390/molecules23030612

**Published:** 2018-03-08

**Authors:** Henri-Pierre Jacquot de Rouville, Claire Kammerer, Gwénaël Rapenne

**Affiliations:** 1 CEMES, Université de Toulouse, CNRS, 31055 Toulouse, France; hpjacquot@unistra.fr (H.-P.J.d.R.); kammerer@cemes.fr (C.K.); 2 Graduate School of Materials Science, Nara Institute of Science and Technology, Takayama 8916-5, Ikoma, Nara, Japan

**Keywords:** nanowheel, wheelbarrow, nanocar, polyaromatic hydrocarbon, triptycene, subphthalocyanine, single molecule, STM, nanocar race

## Abstract

This review article presents our accomplished work on the synthesis of molecular triptycene wheels and their introduction into nanovehicles such as wheelbarrows and nanocars, equipped with two and four wheels, respectively. The architecture of nanovehicles is based on polycyclic aromatic hydrocarbons, which provide a potential cargo zone. Our strategy allowed us to obtain planar or curved nanocars, exhibiting different mobilities on metallic surfaces. Our curved nanocar participated in the first nanocar race organized in Toulouse (France) on 28 and 29 April 2017.

## 1. Introduction

In 2013, in a perspective article on “molecule concept nanocars” [1], Christian Joachim and Gwénaël Rapenne proposed “a yearly competition whereby all nanocar designers would meet together for a few days, similar to the microrobotics contests held around the world.” It took almost four years to find a way to organize such a race, find sponsors, leave the time necessary for competitors to design and synthesize their prototypes, and develop a four-tip scanning tunneling microscope (STM) suitable to host the race. In 2017, six competitors from Austria, Germany, Japan, Switzerland, the United States, and France finally met in Toulouse (France) to compare their designs and strategies to speed up the movement of a single nanocar on a metallic surface. On the 28 April 2017, the starting signal was sounded at exactly 11 a.m. by Dr. Jean-Pierre Launay, emeritus Professor at the University Paul Sabatier (Toulouse) and race commissioner.

The Nanocar Race [2] was seen as a major communication event by the general public. In this review, we will describe why it was a highly challenging scientific event, which was selected to be a part of “Research of the Year 2017” by Chemical Engineering News [3], the scientific journal of the American Chemical Society, as well as the leading French newspaper Le Monde [4]. The last 15 years of research in our group will be detailed, from the design and synthesis of the first molecular wheelbarrow (a platform with two wheels), to the different prototypes of nanocars developed for the nanocompetition.

Nanovehicles are molecular machines able to move efficiently and with some kind of control and directionality at the nanoscale. The first prototypes were technomimetic [5], with a chassis connected to wheels. As pioneers in the field, we proposed using triptycenes [6] to act as rolling elements [7] and synthesized the first nanovehicle with two wheels—a wheelbarrow [8]. Two years after us, Tour’s group reported a family of nanovehicles consisting of a molecular-scale chassis with four [60]-fullerene wheels [9]. Various nanocars were subsequently synthesized by the same group using carboranes [10], ruthenium organometallic complexes [11], and adamantanes [12] as alternative wheels. During that time, we also proposed another wheel candidate, subphthalocyanin [13], which gave very good STM images with direct information about the rolling motion on a metallic surface [14], but unfortunately too unstable to be incorporated in nanovehicles. In the technomimetic family, Feringa et al. proposed a nanovehicle with four motors as wheels [15] and Masson and Hla designed and synthesized the largest car of about 3.5 nm in length. Officially called the Bobcat Nano-Wagon, it had a pseudorotaxane H-shaped frame with four cucurbituril molecules as wheels [16]. It must be noted that in the last example, there was no covalent bond linking the wheels and the axle, but instead a supramolecular charge-transfer interaction.

Like physics, which follows different laws at the nanoscale (quantum physics) as compared to the macroscopic world (Newtonian physics), the design of nanovehicles will not follow the same rules developed for vehicles that surround us in our everyday life. In this way, a new kind of nanovehicle design evolved at the nanoscale. Some nanocars were developed without wheels or motors, and were able to transform an electronic excitation into a controlled movement. Indeed, the teams from Germany, Japan, and Switzerland competed during the Nanocar Race with such nanovehicles. The Japanese team synthesized a molecule similar to a butterfly, consisting of a bisbinaphthyldurene, with two binaphthyl groups (the wings) connected to a central durene spacer [17]. The German team and the Swiss team selected commercially available molecules: 4-acetylbiphenyl [18] and 4′-(*p*-tolyl)-2,2′:6′,2′′-terpyridine [19], respectively. It must be noted that the German nanocar (“the Nano-Windmill”) was composed of four 4-acetylbiphenyl units self-assembled on the surface and stabilized by hydrogen bonds.

We present here our contribution to the field, from the synthesis of a molecular wheelbarrow, the first direct proof of the rolling motion of the wheels and their introduction into four-wheel nanovehicles, to our participation in the first Nanocar Race.

## 2. The First Two-Wheeled and Two-Legged Vehicle: The Molecular Wheelbarrow

The Toulouse team published the design for the first molecule-vehicle in 2002 [7], and it was synthesized in 2003. It was equipped with a chassis, two wheels at the front, two legs, and two handles at the rear, structurally similar to a wheelbarrow [8]. In the case of a macroscopic wheelbarrow, pushing results in the rotation of the wheels. The purpose of this first vehicle was thus to observe the rotational movement of the two front wheels while pushing on the handles and keeping the rear part of its chassis far from the surface, thanks to the two rear molecular legs.

### 2.1. Design

**Figure 1:**
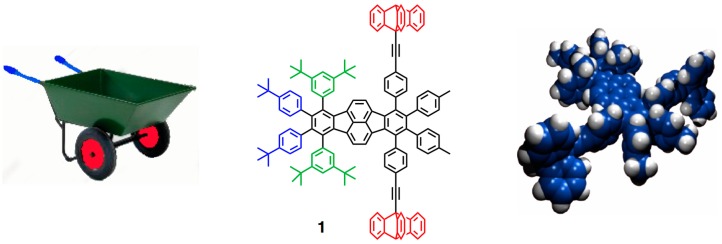
Macroscopic wheelbarrow (**left**), chemical structure of the molecular analogue **1** (**center**), and space-filling model showing its minimum energy conformation (**right**).

Wheelbarrow **1** consists of two legs (3,5-di-*tert*-butylphenyl groups, shown in green in Figure 1) and two three-cogged wheels (triptycene groups, shown in red in Figure 1), which can freely rotate around the axle thanks to the presence of ethynyl spacers [20]. As shown in Figure 1, the skeleton consists of polycyclic aromatic hydrocarbons (PAHs), which due to their rigidity make manipulation by the STM tip easy. As anticipated, the two 3,5-di-*tert*-butylphenyl legs were shown to be held in a conformation in which the phenyl groups are nearly perpendicular to the main aromatic board. This was exploited to lift the chassis, and thus to minimize its interaction with the surface. Moreover, *tert*-butyl groups connected to PAHs are used to increase organic solubility and are easily observed by STM techniques, inducing good contrast in the image. The two 4-*tert*-butylphenyl groups (in blue) play the role of handles for subsequent manipulation using the tip of the microscope.

### 2.2. Synthesis

**Scheme 1:**
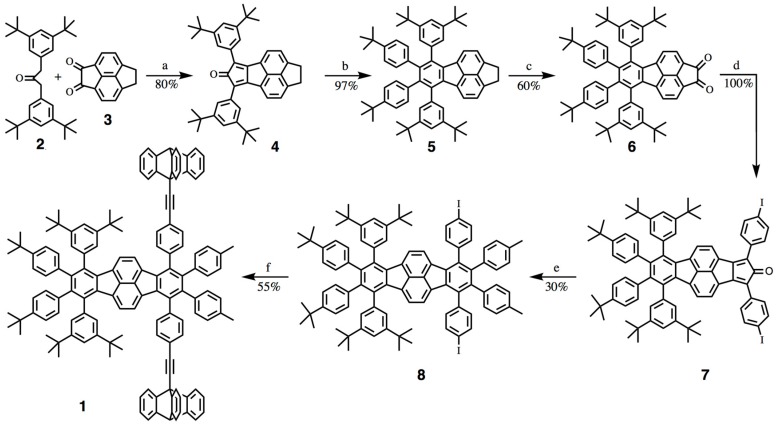
Synthesis of molecular wheelbarrow **1**: reagents and conditions: (**a**) KOH, EtOH, 20 h, Ar, 20 °C; (**b**) di(4-*tert*-butylphenyl)acetylene, diphenylether, 16 h, Ar, reflux; (**c**) (C_6_H_5_SeO)_2_O, chlorobenzene, 62 h, Ar, reflux; (**d**) 1,3-bis(4-iodophenyl)propan-2-one, KOH, EtOH, Ar, reflux; (**e**) di(4-tolyl)acetylene, diphenylether, 16 h, Ar, reflux; (**f**) 9-ethynyltriptycene, Pd(PPh_3_)_4_ 10 mol %, CuI 20 mol %, piperidine-THF (1:1), 24 h, Ar, 20 °C.

The synthesis of the molecular wheelbarrow **1** was achieved in twelve steps, with an overall yield of 2% [21]. As shown in Scheme 1, our strategy used the repetition of a “double Knœvenagel condensation—Diels–Alder reaction” sequence on an α-diketo fragment. The first sequence allowed the connection of the two 3,5-di-*tert*-butylphenyl legs (Step a) and the handles (Step b), while the second one provided the precursor for the connection of the wheels (Steps d and e). Thus, 1,3-di(4-iodophenyl)propan-2-one was selected as partner in this last double Knoevenagel reaction to introduce the iodine centers necessary for the final double Sonogashira coupling, which yielded the molecular wheelbarrow (Step f).

The starting cyclopentadienone **4** was obtained first via a double Knœvenagel reaction of 1,3-bis(3,5-di-*tert*-butylphenyl)propan-2-one **2** with diketopyracene **3** (Step a). The Diels–Alder reaction of cyclopentadienone **4** with di-(4-*tert*-butylphenyl)acetylene (Step b) provided the ethane-bridged precursor **5** in 97% yield. The ^1^H-NMR spectrum clearly showed the expected 2:1 ratio between the different types of *tert*-butyl groups, belonging to the legs and to the handles, respectively. Oxidation of the ethane bridge of the pyracene fragment with benzeneseleninic anhydride (Step c) yielded the α-diketo fragment **6** necessary for the connection of the second axle. This is the key step in our strategy. Aryl halides are introduced at this stage for subsequent cross-couplings, in order to connect the triptycene wheels. The double Knoevenagel condensation with 1,3-di(4-iodophenyl)propan-2-one (Step d) followed by another Diels–Alder reaction with di(4-tolyl)acetylene provided the diiodo intermediate **8** in 30% yield (Step e). The two wheels were then simultaneously covalently attached to the axle via a double coupling of 9-ethynyltriptycene under classical Sonogashira conditions (Step f), affording the molecular wheelbarrow **1** in 55% yield. 

### 2.3. Single Molecule Scanning Tunneling Microscopy Images and Manipulation

**Figure 2:**
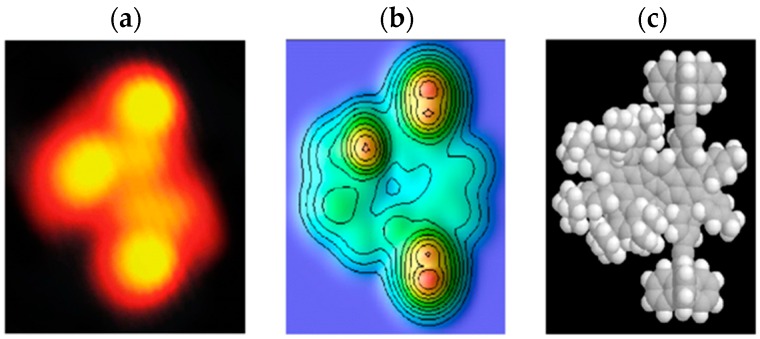
Experimental scanning tunneling microscope (STM) image (3 × 4 nm^2^, V = 0.5 V, I = 0.3 nA) on Cu(100) at 5 K (**a**) and the molecular conformation (**c**) corresponding to the electron scattering quantum chemistry (ESQC) calculated image (**b**).

Such a molecular-scale machine inevitably displays a high level of complexity due to the incorporation of several functionalities within the same molecule. This complexity was reflected in the relatively large molecular weight of the wheelbarrow (1802 g·mol^−1^), which can often lead to problems in the deposition due to the possibility of thermal fragmentations during the sublimation step [22]. In the present case, the required linear wheel axle included two thermally-sensitive triple bonds. However, a few intact molecular wheelbarrows were successfully imaged on a Cu(100) surface, as shown in Figure 2. It leads to a rather complicated STM image, dominated by three intense maxima separated by various weaker patterns, as expected for such a complex chemical structure. The characteristic dimensions, the equivalency of different molecules in the STM images, and comparison with images calculated by electron scattering quantum chemistry (ESQC) method [23] indicated that intact molecular wheelbarrows had been imaged. Manipulation with the STM tip at 5 K resulted in a conformational change in the molecule, which unambiguously confirmed the molecularity of the observed object: the spots obtained were not an assembly of fragments.

Unfortunately, lateral motion of the wheelbarrow could not be achieved. This could be explained by the fact that two wheels may not be enough to allow the motion on the surface of such a large molecule, due to strong coupling. Increasing the number of wheels was proposed to enhance the molecular mobility of nanovehicles, and as a result the design of nanovehicles with four wheels was envisioned.

## 3. The First Rotation of Wheels on a Surface

Wheels are of course one of the key mechanical elements of a vehicle, even on a molecular chassis. At the macroscopic scale, the molecular wheels move the frame slightly away from the supporting surface, in order to lower the lateral diffusion barrier of the molecule-vehicle by reducing the frame–surface electronic interactions. Such decoupling might be achieved using simple molecular legs, although the movement of the molecule on the surface would then cost more energy, as it would require a tilting movement of each molecular leg. As in the macroscopic world, rotation around an axis is thus more energetically favorable.

It was shown that the mechanical motion of a single molecule on a surface can be triggered by the tip apex of the STM [24]. Pushing a molecule with an STM tip was a convenient way to probe the triptycene fragment to act as a wheel at the nanoscale. For that purpose, a prototype composed of two wheels linked to an axle was designed. 

### 3.1. Synthesis and Deposition of a Bis(ethynyltriptycene) as Prototype of a Wheel Dimer

Copper(II)-mediated Glaser homocoupling of 9-ethynyltriptycene fragments—the wheels already used in the molecular wheelbarrow—gave the corresponding dimer of wheels linked via a butadiyne spacer. The latter was a good candidate, since it allowed almost free rotation of the attached fragments [20] in combination with a linear geometry. The ethynyltriptycene dimer has subsequently been studied as a prototype wheel dimer.

**Figure 3:**
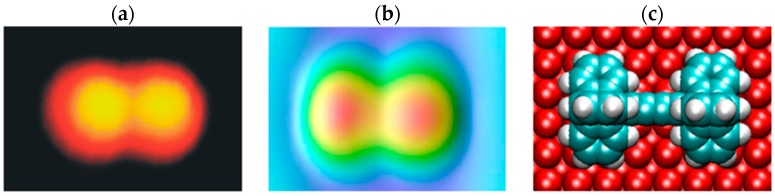
(**a**) Experimental STM image of a wheel dimer on a Cu(110) surface (30 × 30 Å^2^, V = 1 V, I = 0.2 nA) and (**c**) the molecular configuration corresponding to (**b**) the calculated STM image. Adapted from [22], copyright 2006, with permission from Elsevier.

Its deposition on a Cu(110) surface was achieved very cleanly by sublimation under high vacuum [25]. Figure 3 shows the image obtained; each bright lobe corresponds to one triptycene unit, and the observed pair of lobes constitutes the image of a dimer, as confirmed by calculations. No fragmentation or decomposition was observed.

### 3.2. Unidirectional Rotation of the Wheel

For the first time, it was possible to observe the rotation of a wheel on a surface, by working at 5 K under ultra-high vacuum. This was achieved by inducing the translation of the molecule upon pushing it with the STM tip [25]. In order to understand the motion of this molecule in detail, it was particularly important to study the manipulation signal (i.e., the tunneling current recorded at constant tip height during the motion), because the STM images before and after manipulation did not give information about the type of movement between measurements (translational or rotational). 

**Figure 4:**
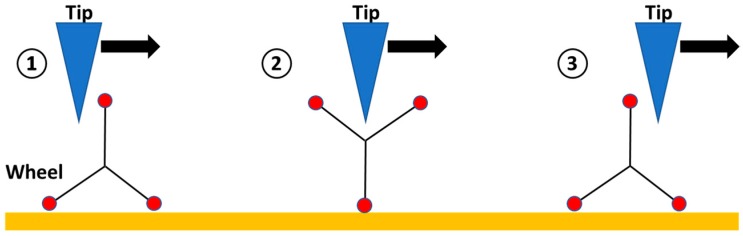
Rotational motion of triptycene wheels. Step 1: the STM tip approaches the molecule; Step 2: the rotation of the wheel starts; Step 3: the STM tip lies on the other side of the wheel after a 120° rotation. Adapted from [25], copyright 2007, with permission from Springer Nature.

The tip was moved across the molecular axle at a constant height, while the tunneling current was recorded. In most cases, it was possible to rotate one wheel, and in a few cases, the rolling of both wheels was observed (as shown in Figure 4). This behavior seems to depend on the precise shape of the tip apex, which in some cases allowed us to address both wheels during the manipulation. This triptycene dimer was the first molecule designed to yield a controlled rotation upon pushing the wheels using the STM tip. With this rolling motion proven, four triptycene units were then integrated into the next generation of nanovehicles, with the aim of increasing mobility on the surface. 

## 4. A Planar Nanocar

**Scheme 2:**
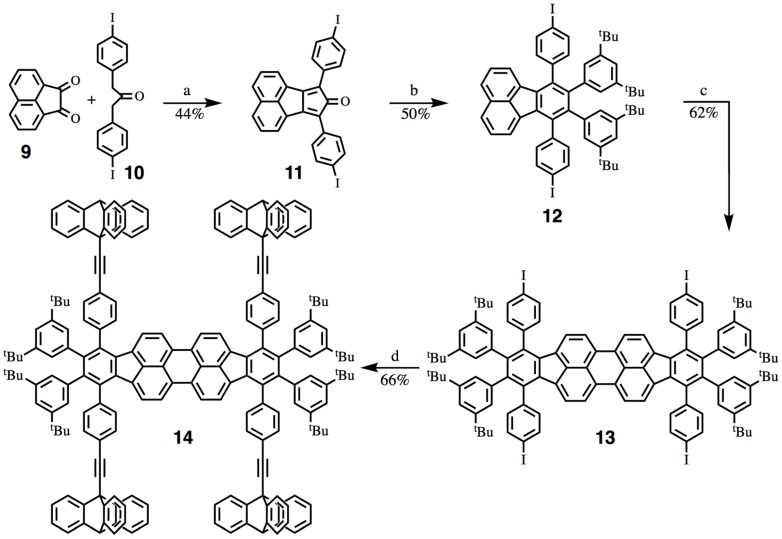
Synthesis of the planar nanocar **14**. (**a**) KOH, MeOH, 2 h, Ar, room temperature (RT); (**b**) 1,2-bis(3,5-di-*tert*-butylphenyl)ethyne, diphenylether, 45 min, 250 °C, microwave (300 W); (**c**) FeCl_3_ 25 eq, CH_2_Cl_2_–CH_3_NO_2_ (9:1), 15 min, Ar, RT; (**d**) 9-ethynyltriptycene 10 eq, Pd(PPh_3_)_4_ 10 mol%, CuI 20 mol%, piperidine-THF (1:1), 6 h, Ar, 80 °C.

For the preparation of nanovehicles containing four wheels (i.e., nanocars), it was necessary to develop the synthesis of a larger chassis based on a perylene unit. This was to sterically accommodate all the wheels on the same molecular structure [26], as the chassis used for molecular wheelbarrow **1** was too short. Therefore, as shown in Scheme 2, our strategy started with the synthesis of di(4-iodophenyl)cyclopentadienone **11** via a double Knœvenagel reaction between acenaphthenequinone **9** and 1,3-di(4-iodophenyl)propan-2-one **10** under basic conditions (Step a). Diels–Alder reaction with 1,2-di(3,5-di-*tert*-butylphenyl)ethyne gave—after aromatization—the half-chassis **12** along with evolution of carbon monoxide (Step b). The half-chassis **12** was subsequently dimerized by using a Scholl-type oxidative coupling with FeCl_3_ as oxidant (Step c). In the last step, the resulting planar chassis **13** was functionalized with four ethynyltriptycenyl wheels via Sonogashira cross-couplings to yield our first prototype nanocar **14** in 9% overall yield.

This nanovehicle was deposited on Au(111). However, due to its planarity and the resulting high interaction of the polyaromatic chassis with the surface, it was not possible to move the vehicle. Thus, we envisioned the design of an alternative molecule-car with a modified chassis, which would undergo less surface coupling.

## 5. A Curved Nanocar

**Scheme 3:**
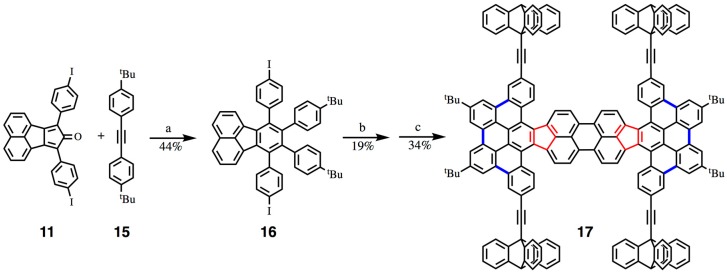
Synthesis of a new curved polyaromatic platform. (**a**) Diphenylether, 45 min, 260 °C, MW (300 W); (**b**) FeCl_3_ 25 eq, CH_2_Cl_2_/CH_3_NO_2_ (9:1), 15 min, Ar, RT; (**c**) 9-ethynyltriptycene 8 eq, Pd(PPh_3_)_4_ 10 mol %, CuI 20 mol %, piperidine/THF (1:1), 6 h, Ar, 80 °C. The additional carbon-carbon bonds obtained in the oxidative coupling are in blue and highlighted in bold, and the cyclopentadiene fragments inducing the curvature are shown in red.

To decrease the interaction of the polyaromatic chassis with the surface, a highly curved chassis was prepared. The synthetic strategy remained identical, but the 3,5-di-*tert*-butylphenyl fragments positioned at both ends were substituted with 4-*tert*-butylphenyl groups [26]. In this case, planarity of contiguous phenyl rings was more easily achieved and dimerization of the half-chassis **16** under Scholl oxidative conditions led to an overcyclized perylene platform bearing six additional C-C bonds, as shown in blue (highlighted in bold) in Scheme 3.

**Figure 5:**
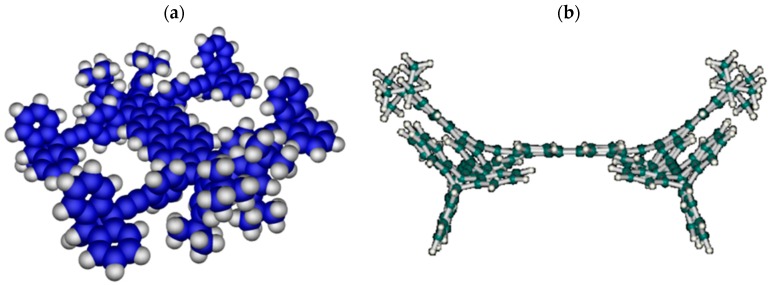
(**a**) CPK (Corey–Pauling–Koltun) model of the nanocar **14** with a planar chassis and (**b**) side view of the optimized geometry of the nanocar **17** with its highly-curved chassis.

The last step consisted of connecting the four triptycene wheels via a quadruple Sonogashira coupling to give the corresponding nanovehicle **17** in 34% yield. Geometric optimization of this second-generation nanovehicle showed that this overcyclized chassis has a curved shape at both ends (Figure 5b). As with fullerenes, this geometry can be explained by the presence of alternating five- and six-membered rings in the polycyclic aromatic hydrocarbon platform. The two five-membered rings are shown in red in Scheme 3. This specificity is of great interest, since it provides an appropriate shape to act as an efficient cargo zone.

The planar and curved nanovehicles have very large differences in their solubilities and their spectroscopic properties. For instance, the color of the planar molecule is pink, while the curved one is dark green; hence, it was called “the green buggy”.

## 6. Participation in the First Nanocar Race: The View from the French Team

On 28 and 29 April 2017, six teams from three continents met in Toulouse to compete with their molecular racers. Both families of nanocars—the technomimetic models incorporating wheels (from Austria, France and the USA) and the smaller and lighter models lacking wheels (from Germany, Japan and Switzerland)—entered the competition [27]. Since the ultra-high-vacuum-low temperature (UHV-LT) STM instrument at Centre d’Elaboration de Matériaux et d'Etudes Structurales (CEMES-CNRS) in Toulouse employs only four tips, four nanocars competed using this microscope. Thus, the Ohio team and the Rice–Graz team were sitting in the same room as the other teams but were remotely piloting their own nanocar, deposited in the STM located at their home university. This presented a further challenge to control a nanocar at the atomic scale from half the world away.

Except for the Rice–Graz team who competed on a silver track, the other nanocars were on gold. The latter surface was originally designated as the common surface for the race, and this was why the official ranking contains two winners, one on gold and one on silver. Each track was 100 nm long and included at least two turns.

### 6.1. Deposition and Imaging of the French Nanocar

**Figure 6:**
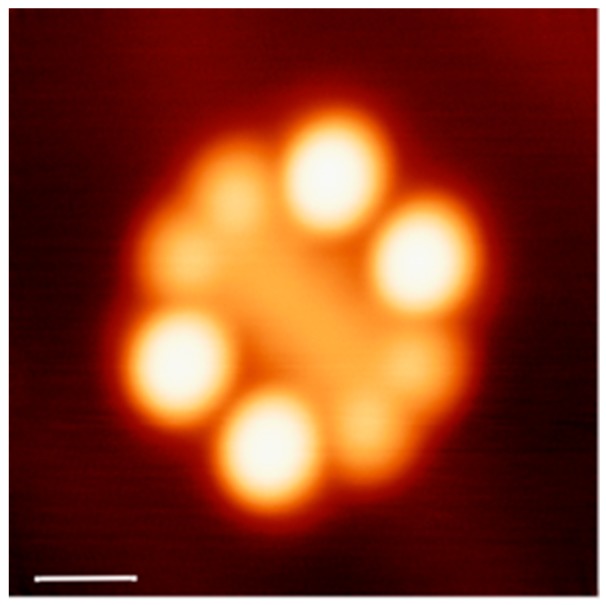
Image of the curved nanocar **17** on Au(111). The four bright spots correspond to the four triptycene wheels (U = 1.1 V, I = 5 pA). Scale bar: 1 nm.

The French nanocar had the advantage of being very robust. It was deposited by sublimation, as with the other nanocars, and was identified on the surface as a long 3 nm molecule. As shown in Figure 6, the triptycene wheels appear as very bright spots when compared with the central polyaromatic core.

### 6.2. The Race and the Official Ranking

On 29 April at 5:00 p.m., after two days and one night of intense efforts, the first ever international nanocar race ended. As mentioned, two teams were ranked first: the Swiss team, racing on Au(111), as stated in the original rules [28] and the Rice–Graz team, racing on Ag(111), since their nanocar appeared to move uncontrollably fast on gold. On Au(111) with the lightest nanocar of the competition (only 42 atoms), the Swiss team arrived first, with a distance of 100 nm covered in six hours [29]. The US team (Ohio) arrived second, with a distance of 43 nm covered. Their nanocar—the Bobcat Nano-wagon—was the largest to participate, with about 650 atoms. Finally, the Nano-Windmill piloted by the German team (Dresden) covered a distance of 11 nm. On Ag(111), the dipolar racer (which consists of one axle with two wheels) was very fast, with a distance of 1000 nm covered in only 29 h [30] but without competitors, since the others competed on Au(111). The characteristics of each team are given in Table 1.

We targeted two strategies to move our nanocar directionally; first, we tried pulling the molecule on the surface, as pushing was forbidden in the rules. The second strategy was to find a way for an electronic excitation to induce the movement. Unfortunately, due to the limited time available both in training and during the race, this propulsion mode failed to drive our nanocar. Only our first strategy was effective, and pulling the molecule with the STM tip allowed us to cover an impressive distance of 25 nm in 3 s. However, we were disqualified from the race as the jury decided not to recognize movement by pulling. We are very satisfied to have participated in the first nanocar race; remembering the motto from the creator of the modern Olympic games, Pierre de Coubertin, which emphasizes that “the importance is to participate”. Moreover, for the first time, we were able to climb a step edge.

We received the “prize of elegance” due to the very smart STM images of our Green Buggy recorded during the race. The Japanese team was not ranked either, as their nanovehicle was unable to recover from a software crash, just one hour into the race. This team was awarded the “fair play” prize.

## 7. Conclusions and Perspectives

In summary, we have presented the design and synthesis of polyaromatic hydrocarbons conceived by analogy with macroscopic wheels, wheelbarrows, and nanocars. Concerning the nanocar race, we had confirmation that the smallest molecules completed the track distance first (the Swiss molecule-car had only 42 atoms and is commercially available). This is partly because smaller was better for easily and rapidly moving on the surface.

The first nanocar race was a real success, with a worldwide audience following the event on the internet, broadcasted live, night and day, with more than 100,000 viewers at peak times. We now have experience in organizing such an event, which may have a second edition around 2021. All the competitors went home with new ideas that will make them more competitive next time, and we can assume that new participants will enter. One could imagine organizing semi-finals, test selections to qualify for the final or to run the race at a higher temperature, in order to have a faster event. It has already been shown that control of motion is possible at higher temperatures on some semi-conductive surfaces, such as SmSi [31,32] or SiB [33]. Furthermore, the question of whether we need to create different categories will be addressed, in order to give everybody the same chance (e.g., dividing the nanovehicles by the number of atoms or by the molecular mass). We could also organize a different race, for example, moving the nanocar with cargo on its backbone. This would be similar to the “waiter’s race” (“course de garçons de café” in French) created in Paris (France), which tests the speed of a waiter carrying a loaded tray without tipping it over [34].

## Figures and Tables

**Table 1:** The six registered participants and their respective nanocars, with their characteristic parameters: country, team leader, landing surface, and number of atoms of their nanovehicle (with the corresponding chemical formula). In red, the parameters outside of the original rules [28] but accepted by the jury.

	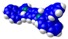	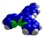	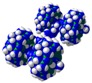	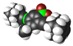	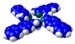	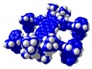
	MANA NIMS	Nano Dragster	Nano-Wagon	Dipolar Racer	Nano-Windmill	Green Buggy
Country	Japan	Switzerland	USA	Austria & USA	Germany	France
**Team leader**	Waka Nakanichi	Rémy Pawlack	Saw Wai Hla & Eric Masson	Leonhard Grill & James Tour	Francesca Moresco	Gwénaël Rapenne
**Surface**	Au(111)	Au(111)	Au(111)	Ag(111)	Au(111)	Au(111)
**Number of atoms**	88 atoms (C_50_H_34_O_4_)	42 atoms (C_22_H_17_N_3_)	642 atoms (C_244_H_222_N_120_0_56_)	72 atoms (C_32_H_36_N_2_O_2_)	108 atoms (C_56_H_48_O_4_)	300 atoms (C_184_H_116_)
